# Biochanin A inhibits lung adenocarcinoma progression by targeting ZEB1

**DOI:** 10.1007/s12672-022-00601-2

**Published:** 2022-12-13

**Authors:** Jianjun Li, Yaqi Kou, Xiaohan Zhang, Xuechun Xiao, Yang Ou, Lixia Cao, Min Guo, Chunchun Qi, Zhaoyang Wang, Yuxin Liu, Qiuying Shuai, Hang Wang, Shuang Yang

**Affiliations:** 1 Tianjin Key Laboratory of Tumor Microenvironment and Neurovascular Regulation, Medical College of Nankai University, 300071 Tianjin, China; 2 Department of Pulmonary and Critical Care Medicine, The First Affiliated Hospital of Soochow University, 215006 Suzhou, China; 3 Institute of Transplantation Medicine, Nankai University, 300071 Tianjin, China; 4 Medical College of Nankai University, 94 Weijin Road, 300071 Tianjin, China

**Keywords:** Lung adenocarcinoma, Biochanin A, ZEB1, Chemosensitization, Proteasomal ubiquitination

## Abstract

Lung adenocarcinoma is the major subtype of lung cancer, accounting for approximately 40% of lung cancers. During clinical treatment, the emergence of chemotherapy resistance seriously affects the effectiveness of treatment. Thus, finding new chemotherapeutic sensitizers is considered to be one of the effective solutions. Biochanin A, as a naturally occurring isoflavone, has been demonstrated to exhibit anticancer effects in various tumors. However, the potential mechanisms of Biochanin A to inhibit tumor development have not been clarified. In the present study, we found that the combinational treatment of cisplatin and Biochanin A exhibited strong synergistic repression on lung adenocarcinoma growth and progression in vitro and in vivo. Considering that epithelial–mesenchymal transition (EMT) is recognized to be associated with both chemoresistance and metastasis, we examined the EMT-related markers and found that Biochanin A could specifically inhibit the expression of ZEB1. Importantly, Biochanin A chemosensitizes lung adenocarcinoma and inhibits cancer cell metastasis by suppressing ZEB1. At the molecular level, Biochanin A affects the stability of ZEB1 protein through the deubiquitination pathway and thereby influences the progression of lung adenocarcinoma. In conclusion, our finding elucidates the potential efficacy of Bichanin A as a chemosensitizer and provides new strategy for the chemotherapy of advanced lung adenocarcinoma.

**Supplementary Information:**

The online version contains supplementary material available at 10.1007/s12672-022-00601-2.

## Introduction

Non-small cell lung cancer (NSCLC) accounts for 85–95% of all lung cancer cases, which is a leading cause of death world-wide [1]. Lung adenocarcinoma is the most common subtype of NSCLC, with an increasing incidence and mortality rate over the past decades [2, 3]. For the treatment of lung adenocarcinoma, chemotherapy is an effective way to relieve the aggressive symptoms and prolong the survival of patients with advanced disease [4]. However, the initial robust benefit of this treatment is frequently compromised by chemoresistance [5, 6], which is associated with a 5-year survival rate of less than 7% [7]. As an approach, finding high-efficiency and low-toxicity chemosensitizers may improve the effect of chemotherapy and prolong the life of lung adenocarcinoma patients.

In recent years, naturally occurring phytochemicals have been widely applied to prevent and treat human cancers [8]. Biochanin A (5,7-dihydroxy-4′-methoxy-iso-flavone,) is a natural isoflavonoid phytoestrogen that is most commonly found in legumes, particularly in the red clover [8]. A growing body of evidences have demonstrated that Biochanin A performs important roles in preventing human cancer development and progression. For example, Biochanin A induces S phase arrest and apoptosis in lung cancer cells [9]. Biochanin A also synergistically enhances the antiproliferative and apoptotic effects of sorafenib in hepatocellular carcinoma cells [10]. Of note, liposomes that contain Biochanin A and doxorubicin reverse chemoresistance in colon cancer [11]. However, the potential mechanisms of Biochanin A in the regulation of cancer chemoresistance have not been elucidated yet.

Zinc-finger E-box binding homeobox 1 (ZEB1) is a key regulator of epithelial-mesenchymal transition (EMT), which is a biologic process that allows a polarized epithelial cell to assume a mesenchymal cell phenotype, including enhanced migratory capacity, invasiveness and elevated resistance to apoptosis, etc. [12]. ZEB1 can directly inhibit the expression of E-cadherin by binding its zinc finger structure to E_2_-boxes in the promoter region of the E-cadherin gene, thus initiating the process of EMT [13–15]. ZEB1 knockdown has been implied to inhibit the invasive and metastatic ability of tumor cells [14, 15]. For example, ZEB1 knockdown inhibits Hoxd9-induced migration and invasion of hepatocellular carcinoma cells [16]. miR-200 family members can repress ZEB1 expression and thus inhibit the capacity of ovarian cancer cells to undergo migration and invasion [17]. ZEB1 has also been shown to be associated with tumorigenesis and chemoresistance in various human cancers [18–20].

In the present study, we demonstrated that Biochanin A exerts a chemosensitizing effect by targeting ZEB1 in vivo and in vitro. Mechanistically, Biochanin A affects the stability of ZEB1 protein through the proteasomal ubiquitination degradation pathway. These findings collectively uncover the potential mechanism of Biochanin A/ZEB1 signaling to inhibit lung adenocarcinoma development, which provides a new strategy for the treatment of advanced lung adenocarcinoma.

## Results

### Biochanin A chemosensitizes lung adenocarcinoma to cisplatin

**Fig. 1:**
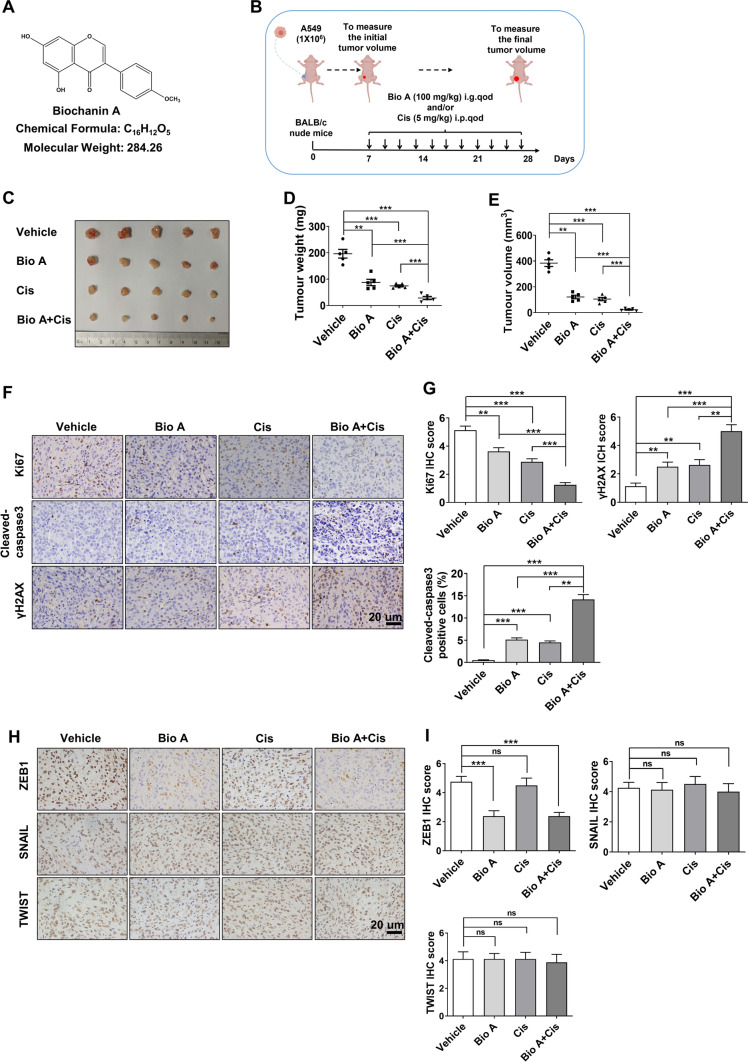
Biochanin A chemosensitizes lung adenocarcinoma to cisplatin. **A** The chemical structure of Biochanin A. **B** Experimental design of tumor xenograft in nude mice by treatment with Biochanin A and/or cisplatin. **C** Tumors from mice with treatment of Biochanin A and/or cisplatin are shown.  Approximate tumor weight (**D**) and tumor volume (**E**) were analyzed. **P < 0.01, ***P < 0.001 vs. the respective control by an unpaired Student’s *t*-test. **F, G** The expression of Ki67, cleaved-caspase 3 and γH2AX were measured by immunohistochemistry analysis. Scale bars, 20 μm. **P < 0.01, ***P < 0.001 vs. the respective control by an unpaired Student’s *t*-test. **H**, **I** The expression of EMT-TFs (ZEB1, SNAIL and TWIST) were measured by immunohistochemistry analysis. Scale bars, 20 μm. ***P < 0.001, vs. respective control by an unpaired Student’s *t*-test

To investigate whether Biochanin A could regulate the chemosensitivity in lung adenocarcinama, we performed a tumor xenograft experiment in vivo. To do so, A549 cells were subcutaneously injected into BALB/c nude mice in the presence or absence of Biochanin A and/or cisplatin (Fig. 1A, B). The results showed that either Biochanin A (100 mg/kg) or cisplatin (5 mg/kg) treatment suppressed the tumor volume and weight by 50–60%; however, a stronger inhibition of tumor growth was achieved by the combinatorial treatment of Biochanin A and cisplatin, with over 80% antitumor efficacy (Fig. 1C–E). The immunohistochemical staining of Ki67, cleaved-caspase3 and γH2AX also demonstrated the synergistic inhibition in cell proliferation and the promotion of cell apoptosis and DNA damage response in tumors with the combinatorial treatment of Biochanin A and cisplatin (Fig. 1F, G). Changes of mice body weight and toxic pathological in the heart, liver, spleen, lung, and kidney were not evident (Additional file 1: Figure S1A and B). These observations collectively implied a chemosensitizing potency of Biochanin A in lung adenocarcinoma in vivo.

Considering that EMT have been reported to be highly associated with chemotherapeutic resistance in lung cancer [21–23], we moved to validate whether the EMT-inducing transcription factors (EMT-TFs) including ZEB1, SNAIL and TWIST were involved in Biochanin A-regulated chemosensitivity. The results of immunohistochemical staining showed that ZEB1 expression was significantly downregulated by Biochanin A regardless of cisplatin treatment, while the alternation of SNAIL and TWIST was not as evident (Fig. 1H, I). These observations indicated that Biochanin A might exert its effect on chemosentizition by specifically inhibiting ZEB1 expression in lung adenocarcinoma.

### Biochanin A chemosensitizes lung adenocarcinoma through downregulation of ZEB1

To further investigate the relationship between Biochanin A and ZEB1 in vitro, A549 and H1299 cells were treated with different concentrations of Biochanin A. Immunoblotting assay showed that the expression of ZEB1 was suppressed by Biochanin A in a dose-dependent manner (Additional file 1: Figure S2A, B). Moreover, treatment with 100 µM Biochanin A resulted in significant increase in E-cadherin (an epithelial marker) expression but decrease in Vimentin and N-cadherin (mesenchymal markers) expression (Additional file 1: Figure S2C, D). Similar results were further observed by immunohistochemical staining of E-cadherin, Vimentin and N-cadherin (Additional file 1: Figure S2E, F), confirming that Biochanin A induces ZEB1 downregulation and thus affects its subsequent events, such as EMT.

**Fig. 2:**
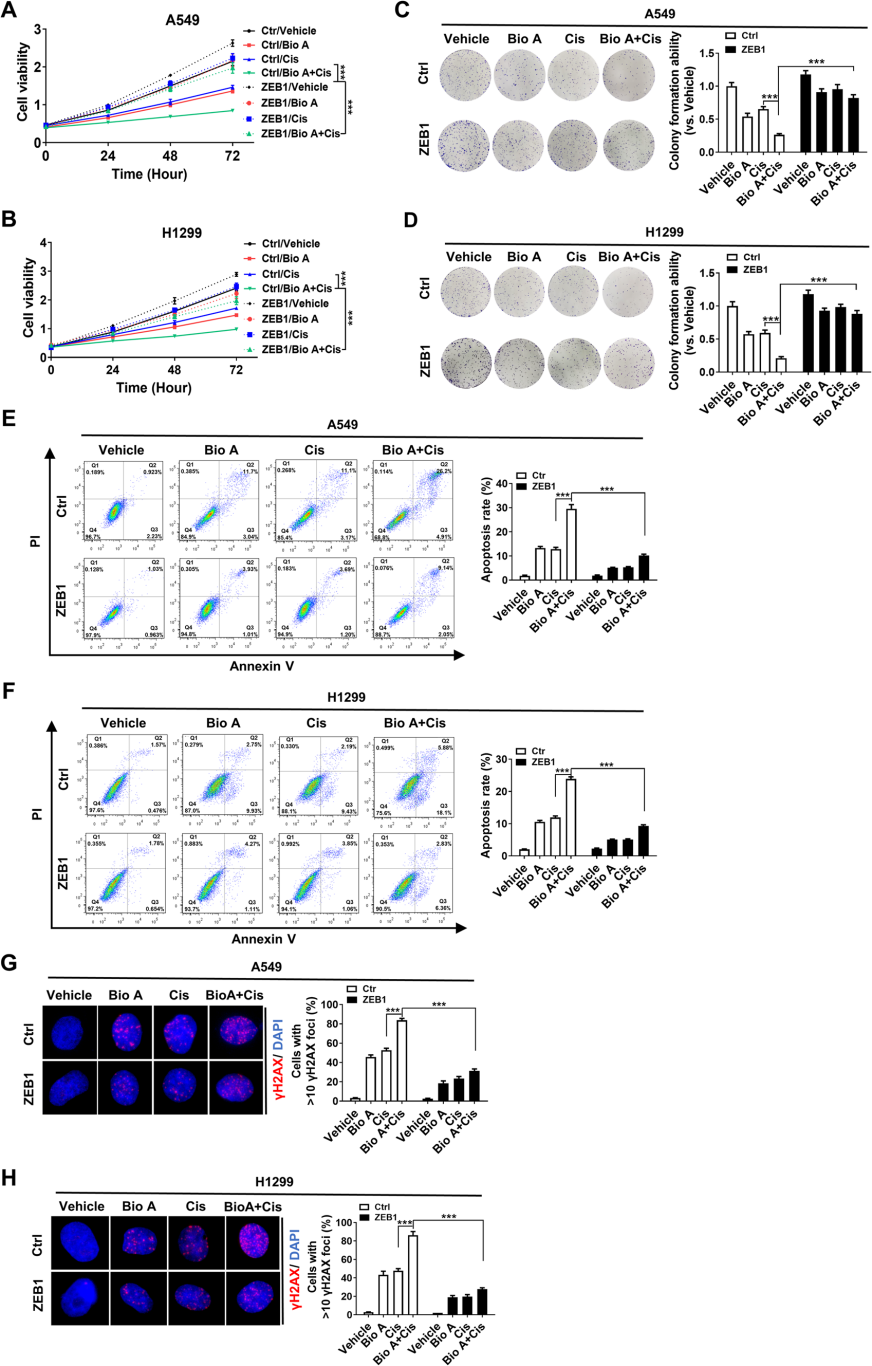
Biochanin A chemosensitizes lung adenocarcinoma in a ZEB1-dependent manner in vitro. Cell viability assay in ZEB1-expressiong A549 (**A**) and H1299 (**B**) cells by treatment with Biochanin A and/or cisplatin. ***P < 0.001 by two-way ANOVA with Sidak correction for multiple comparisons. Colony formation assay in ZEB1-expressiong A549 (**C**) and H1299 (**D**) cells by treatment with Biochanin A and/or cisplatin. Cell apoptotic assay of Annexin V/PI staining in ZEB1-expressiong A549 (**E**) and H1299 (**F**) cells by treatment with Biochanin A and/or cisplatin. Immunofluorescence staining of γH2AX in ZEB1-expressiong A549 (**G**) and H1299 (**H**) cells by treatment with Biochanin A and/or cisplatin. *P < 0.05, **P < 0.01, ***P < 0.001 vs. respective control by an unpaired Student’s *t*-test

In addition, ZEB1 was overexpressed in A549 and H1299 to establish the stable cell lines (Additional file 1: Figure S3A, B), followed by treatment with cisplatin in the presence or absence of Biochanin A. The results of CCK8 assay showed that addition of Biochanin A remarkedly reduced the cell viability of Ctrl/A549 cells in response to cisplatin; however, this effect was attenuated by ectopic expression of ZEB1 in ZEB1/A549 cells (Fig. 2A). Similar results were also obtained in H1299 cells (Fig. 2B). Moreover, the analysis of colony formation (Fig. 2C, D), cell apoptosis (Fig. 2E and F; Additional file 1: Figure S4A–S4D) and γH2AX foci formation (Fig. 2G, H; Additional file 1: Figure S4E, F) demonstrated that Biochanin A treatment enhanced the chemosentivity to cisplatin in lung adenocarcinoma cells, which in effect was mediated through downregulation of ZEB1.

**Fig. 3:**
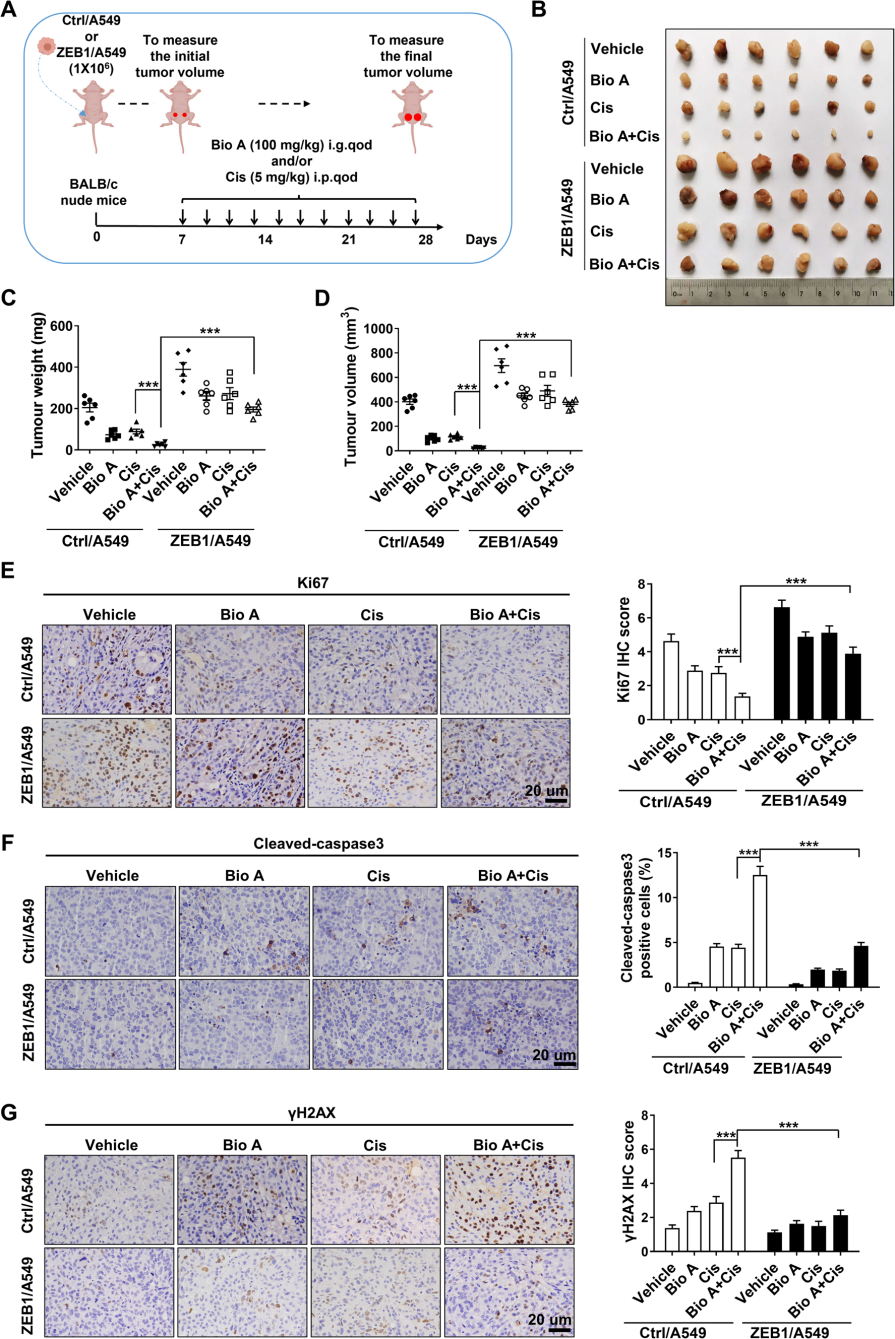
Biochanin A chemosensitizes lung adenocarcinoma in a ZEB1-dependent manner in vivo. **A** Experimental design of ZEB1-expressiong A549 tumor xenograft in nude mice by treatment with Biochanin A and/or cisplatin. **B** Tumors from mice by treatment with Biochanin A and/or cisplatin are shown. Approximate tumor weight (**C**) and tumor volume (**D**) were analyzed. ***P < 0.001 vs. the respective control by an unpaired Student’s *t*-test. The expression of Ki67 (**E**), cleaved-caspase 3 (**F**) and γH2AX (**G**) were measured. Scale bars, 20 μm. ***P < 0.001 vs. respective control by an unpaired Student’s *t*-test

Consequently, to confirm these results in vivo, the lung adenocarcinoma xenograft model was established by subcutaneous injection of Ctrl/A549 and ZEB1/A549 cells into BALB/c nude mice, followed by treatment with Biochanin A and/or cisplatin (Fig. 3A). We observed that either Biochanin A or cisplatin treatment suppressed the tumor growth, while the combinational treatment of Biochanin A and cisplatin had a synergistic inhibitory effect in mice carrying Ctrl/A549 tumors (Fig. 3B–D). Notably, ectopic expression of ZEB1 significantly abolished the inhibition in tumor growth by Biochanin A and/or cisplatin (Fig. 3B–D). The immunohistochemical staining of Ki67 (Fig. 3E), cleaved-caspase3 (Fig. 3F) and γH2AX (Fig. 3G) also confirmed that ZEB1 downregulation contributed to Biochanin A-induced chemosensitization to cisplatin, highlighting that Biochanin A plays an important role in the regulation of chemosensitivity of lung adenocarcinoma through ZEB1 in vitro and in vivo.

### Biochanin A inhibits lung adenocarcinoma metastasis through downregulation of ZEB1

**Fig. 4:**
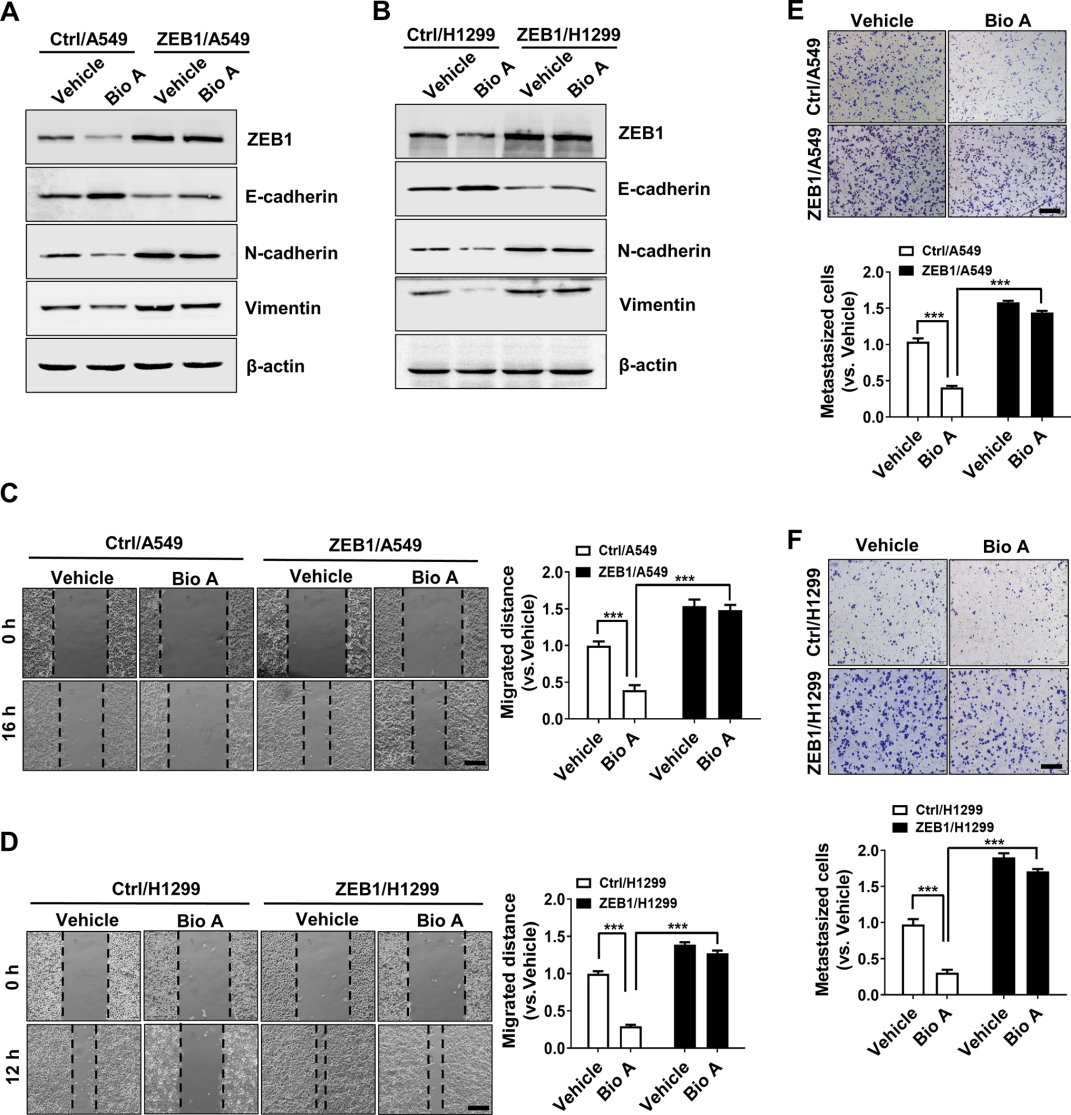
Biochanin A inhibits lung adenocarcinoma metastasis in a ZEB1-dependent manner in vitro. Immunoblotting of EMT markers in A549 (**A**) and H1299 (**B**) cells in the presence or absence of Biochanin A. Wound-healing assay in ZEB1-expressing A549 (**C**) and H1299 (**D**) cells in the presence or absence of Biochanin A. Scale bars, 100 μm. ***P < 0.001 vs. respective controls by an unpaired Student’s *t*-test. Transwell assay in ZEB1-expressing A549 (**E**) and H1299 (**F**) cells in the presence or absence of Biochanin A. Scale bars, 100 μm. ***P < 0.001 vs. respective controls by an unpaired Student’s *t*-test

Considering that ZEB1 functions as a key EMT transcriptional factor in tumor invasion and metastasis, we moved to validate whether Biochanin A affects cancer metastasis via the regulation of ZEB1 in lung adenocarcinoma. As shown in Fig. 4A, B, treatment with Biochanin A increased the expression of E-cadherin but decreased the expression of Vimentin and N-cadherin in A549 and H1299 cells; however, this effect was significantly weakened by ectopic expression of ZEB1. Furthermore, wound-healing (Fig. 4C, D) and transwell (Fig. 4E, F) assays demonstrated that Biochanin A inhibited cell migration and invasion in lung adenocarcinoma cells, which was mediated through the downregulation of ZEB1 expression.

**Fig. 5:**
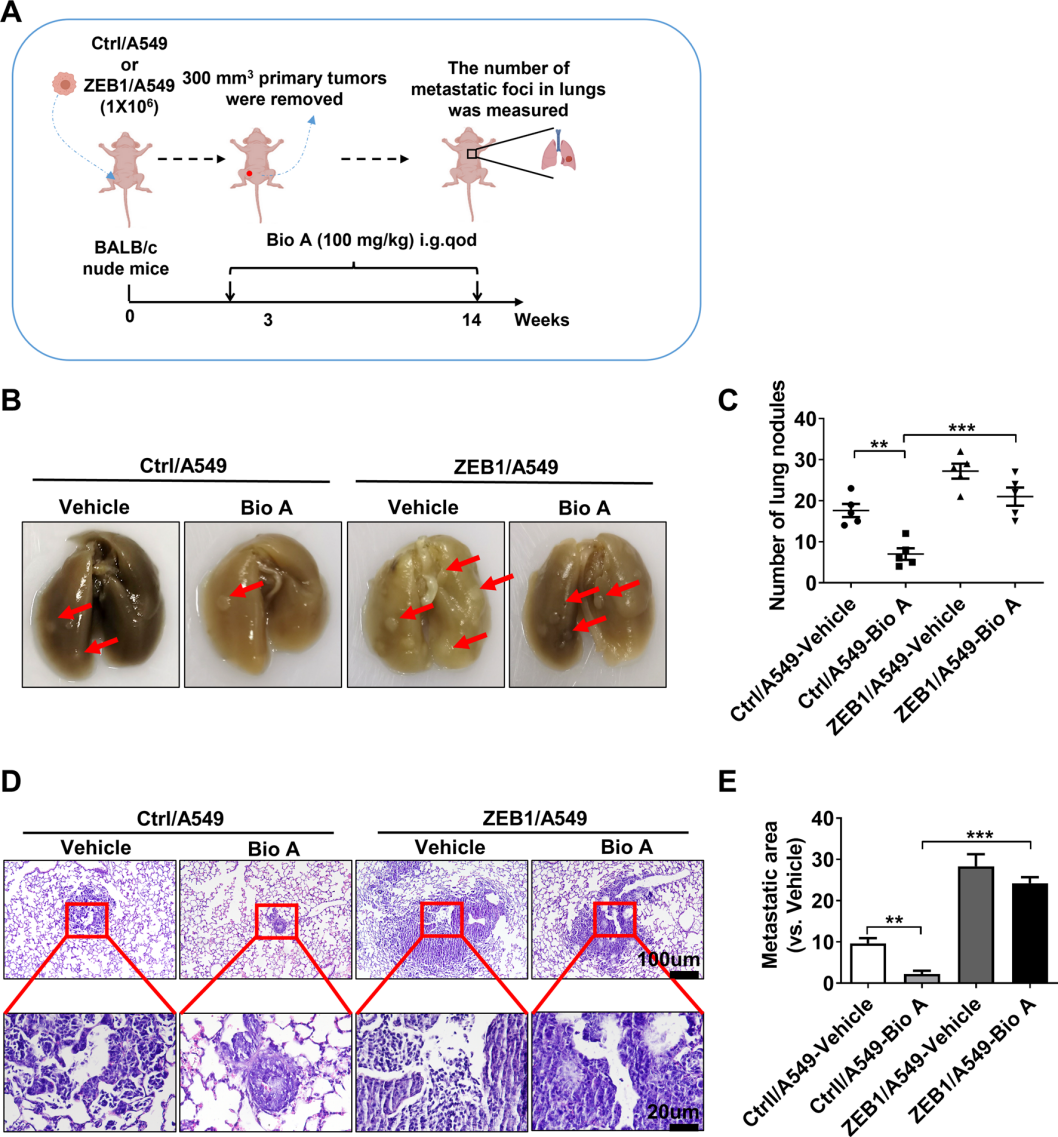
Bio A inhibits lung adenocarcinoma metastasis in a ZEB1-dependent manner in vivo. **A** Experimental design of metastatic tumor xenograft in nude mice by treatment with Biochanin A. Representative images (**B**) and quantification (**C**) of the metastatic lung nodules from mice that were injected with ZEB1-expressing A549 cells by treatment with Biochanin A. **P < 0.01, ***P < 0.001 vs. the respective control by an unpaired Student’s *t*-test. Representative images (**D**) and quantification (**E**) of the metastatic area from mice that were injected with ZEB1-expressing A549 cells by treatment with Biochanin A. **P < 0.01, ***P < 0.001 vs. the respective control by an unpaired Student’s *t*-test

Next, we investigated the effects of Biochanin A/ZEB1 on cancer cell metastasis in a xenograft metastasis model. As shown in Fig. 5A, Ctrl/A549 and ZEB1/A549 (2 × 10^6^) cells were subcutaneously injected into nude mice. When the volume of primary tumors reached 300 mm^3^, the primary tumors were surgically resected and the mice were treated with Biochanin A for 12 weeks. The results showed that the number of metastatic foci in lungs was significantly reduced by Biochanin A treatment in mice carrying Ctrl/A549 tumors (Fig. 5B–E). However, this effect was attenuated in mice carrying ZEB1-expressing tumors, confirming that Biochanin A treatment decreases lung adenocarcinoma metastasis through the regulation of ZEB1 in vivo.

### Biochanin A regulates ZEB1 protein stability via proteasomal ubiquitination degradation

**Fig. 6:**
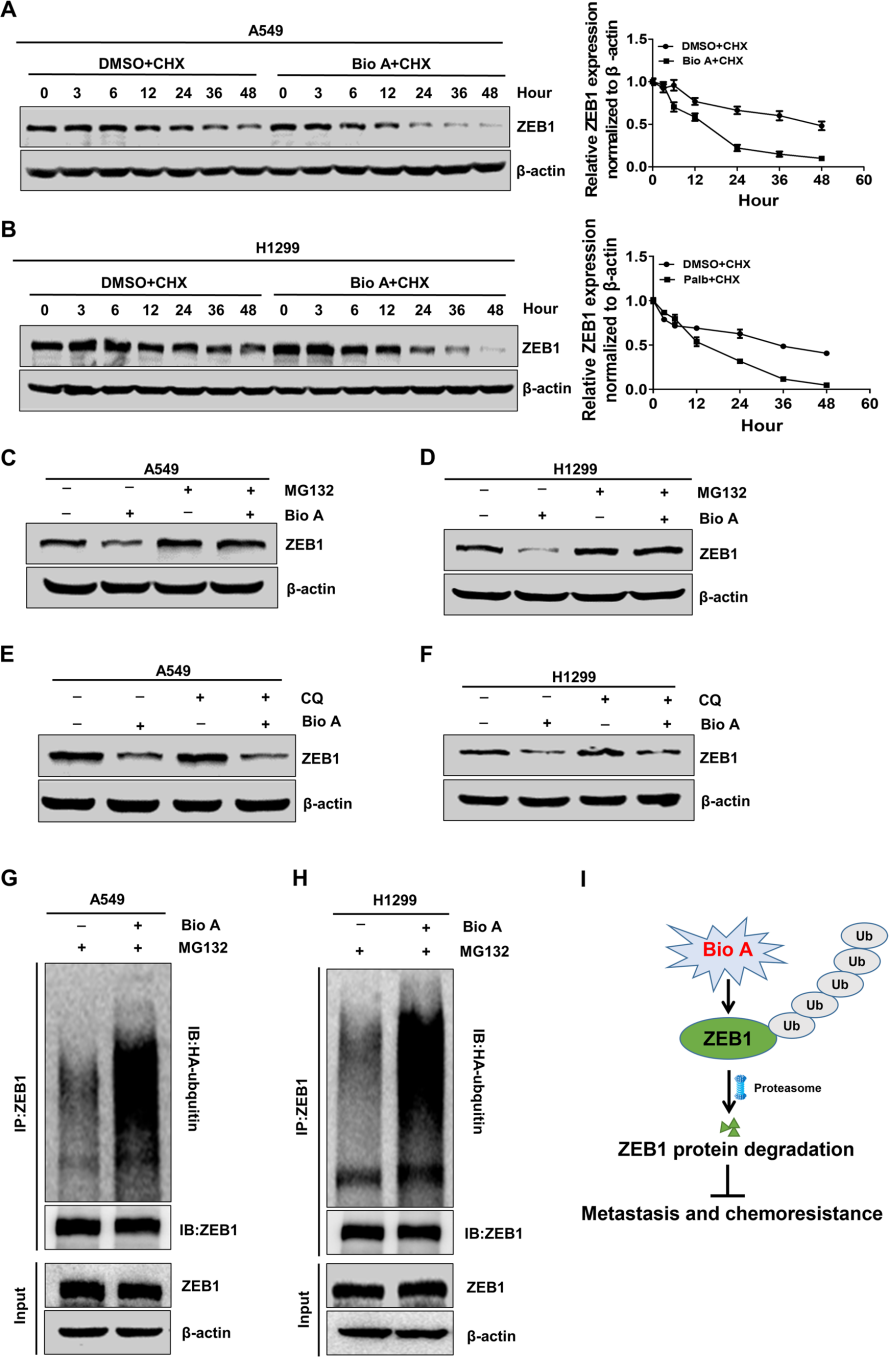
Biochanin A regulates ZEB1 protein stability via proteasomal ubiquitination degradation. CHX pulse-chase analysis of ZEB1 protein stability in A549 (**A**) and H1299 (**B**) cells by treatment with Biochanin A at the indicated time points. Immunoblotting of ZEB1 protein expression in A549 (**C**) and H1299 (**D**) cells by treatment with MG132 in the presence or absence of Biochanin A. Immunoblotting of ZEB1 protein expression in A549 (**E**) and H1299 (**F**) cells by treatment with CQ in the presence or absence of Biochanin A. Co-immunoprecipitation analysis of ZEB1 protein ubquitination in A549 (**G**) and H1299 (**H**) cells by treatment with MG132 in the presence or absence of Biochanin A. (**I**) A working model to illustrate that Biochanin A inhibits chemoresistance and metastasis in lung adenocarcinoma by inducing ZEB1 protein degradation via the ubiquitin-proteasome pathway

Next, we moved to investigate the underlying mechanism of ZEB1 downregulation by Biochanin A in lung adenocarcinoma. We found that treatment with Biochanin A did not alter the mRNA expression of ZEB1 in A549 and H1299 cells (Additional file 1: Figure S5A, B). Considering that Biochanin A inhibited ZEB1 expression at the protein level, we further examined the stability of ZEB1 protein in response to Biochanin A by immunoblotting. To do so, A549 and H1299 cells were treated with a protein synthesis inhibitor cycloheximide (CHX) for 48 h in the presence or absence of Biochanin A. The results showed addition of Biochanin A markedly reduced the protein stability of ZEB1 (Fig. 6A and B). It has been well established that the ubiquitin-proteasome and autophagy-lysosome pathways play predominant roles in the regulation of protein degradation in eukaryotes [24]. A549 and H1299 cell lines were then treated with a proteasome inhibitor MG132 and a lysosomal inhibitor chloroquine (CQ) in the presence or absence of Biochanin A, respectively. The results demonstrated that Biochanin A-induced protein degradation of ZEB1 was significantly inhibited by MG132, while the effect of CQ was not as evident (Fig. 6C-F). Taken together with the results of ubiquitination assay showing that Biochanin A treatment enhanced the ubiquitination levels of ZEB1 protein in A549 and H1299 cells (Fig. 6G and H), these data collectively illustrated that Biochanin A induces ZEB1 protein degradation via the ubiquitin-proteasome pathway.

## Discussion

For patients with lung adenocarcinoma receiving chemotherapy, the most significant obstacle to the effectiveness of chemotherapy is the emergence of chemoresistance. Currently, the main causes of chemoresistance still remains obscure. Therefore, it is of great practical importance to find new chemotherapeutic sensitizers to improve the sensitivity of chemotherapy. Biochanin A, as a natural product, has been reported to inhibit the proliferation of several human cancers. However, the chemosensitization potential of Biochanin A and its underlying mechanisms in lung adenocarcinoma are unknown. In the present study, we demonstrated that Biochanin A performs a strong chemosensitizing effect on lung adenocarcinoma. Moreover, this effect was achieved by inhibiting the expression of ZEB1 through the proteasomal ubiquitination pathway (Fig. 6I).

It has been reported that Biochanin A exerts anticancer effects through different pathways. For example, Biochanin A inhibits cell migration and invasion in a dose-dependent manner in malignant melanoma, leading to the elevated expression of key proteins in the NF-κB and MAPK signaling pathways [25]. In human osteosarcoma, Biochanin A reduces cancer cell growth via the mitochondrial-involved apoptosis [8]. Biochanin A can also be used in combination with other drugs, such as ginsenoside Rh2 or BRAF inhibitor SB590885, to inhibit the proliferation of breast or hepatocellular cancers [26, 27]. Here, we extended the study that Biochanin A performs a potent chemosensitization activity in lung adenocarcinoma in vitro and in vivo. At the molecular level, Biochanin A decreases the protein stability of ZEB1 via the proteasomal ubiquitination degradation pathway, which is consistent with the notion that ectopic ZEB1 confers chemoresistance in human cancers [28, 29].

It is well established that the dysfunction of EMT is correlated with cancer cell metastasis as well as therapeutic resistance [30]. As a key EMT-TF, ZEB1 regulates the crosstalk of EMT and chemoresistance in a variety of tumors. For example, ZEB1 promotes chemoresistance to cisplatin by suppressing SLC3A2 in ovarian cancer [31]. Targeted suppression of ZEB1 also improves the sensitivity to doxorubicin and thus inhibits the cell migration in hepatocarcinoma [32]. In this study, our results further demonstrated that inhibition of ZEB1 expression by Biochanin A significantly enhances the sensitivity of lung adenocarcinoma cells to cisplatin in vitro and in vivo. However, the expression of TWIST and SNAIL were not affected, highlighting that Biochanin A might perform a specific inhibitory effect on the malignant progression of lung adenocarcinoma through ZEB1. In line, Zhang et al. has previously reported that only ZEB1, but not TWIST and SNAIL, confers radioresistance in breast cancer [33]. These findings point to functional differences of EMT-TFs, showing that ZEB1 may exert different tumorigenic functions (e.g., chemoresistance and EMT) that are not necessarily interrelated.

Of note, the ubiquitin-proteasome system is the main protein-degradation pathway in eukaryote cells, which is responsible for more than 80% of protein degradation [34–36]. Several recent studies have identified the posttranslational modifications for the regulation of ZEB1 protein levels in cancer development. For example, Zhou et al. reported that the knockdown of ubiquitin-specific protease 51 (USP51), which is a deubiquitinase, attenuates cisplatin resistance in lung cancer through the ubiquitination of ZEB1 [37]. Moreover, a E3 ligase TRIM26 could promote the degradation of ZEB1 by protein ubiquitination, resulting in the inhibition of cell proliferation and metastasis in hepatocellular carcinoma [38]. Consistent with these findings, we showed that the proteasome inhibitor MG132, but not the lysosome inhibitor CQ, significantly suppressed the degradation of ZEB1 protein by Biochanin A treatment in lung adenocarcinoma cells, demonstrating the ubiquitination-mediated protein stability is involved in this process. However, the particular mechanisms of ZEB1 regulation by Biochanin A needs further exploration.

In conclusion, we have demonstrated an alternative role of Biochanin A in regulating lung adenocarcinoma cell proliferation and metastasis that supplements the deubiquitination and stabilization of ZEB1. Our finding thus highlights the potential efficacy of Biochanin A as a chemosensitizer and provides a promising strategy for the chemotherapy of advanced lung adenocarcinoma.

## Materials and methods

### Cell culture

A549 and H1299 cell lines from the American Type Culture Collection were maintained in RPMI-1640 supplemented with 10% fetal bovine serum (FBS), 100 U/ml penicillin/streptomycin at 37 °C in 5% CO2.

### Reagents

Biochanin A was purchased from Sigma, dissolved in DMSO as a stock concentration of 100 mM, and stored at -20 °C. Cisplatin was purchased from TargetMol.

### Lentiviral expression systems

The human cDNA fragment encoding ZEB1 was prepared by PCR and cloned into pLV-EF1α-MCS-IRES-Bsd (Biosettia). The lentiviruses were generated by transfecting subconfluent 293T cells with lentiviral vectors and packaging plasmids using the Lipofectamine 3000 reagent (Invitrogen). The primer sequences are listed in Additional file 1: Table S1.

### Immunoblotting assay

Preparation of the total cell extracts and immunoblotting was performed as previously described [39]. The antibodies used in the experiment are listed in Table S2.

### Wound-healing assay

Cells were allowed to grow to full confluence. The confluent monolayer of cells was scraped using a 10 µl tip. The complete medium was replaced with serum-free medium, followed by treatment with Biochanin A. A light microscope (Olympus) and ImageJ software were used to calculate the migration distance.

### Transwell assay

Cells were placed into the upper chamber in basal medium without FBS, while the lower layer was filled with DMEM containing 10% FBS. After 16 h, the nonmigrated cells were scraped with a cotton swab, whereas the migrated cells were then fixed with 20% methanol and stained with 0.5% crystal violet. The stained cells were counted and photographed under a light microscope (Olympus).

### Cell viability

Briefly, 3000 cells/well were plated into 96-well, grown for overnight, treated with cisplatin (8 µM) and/or Biochanin A (100 µM) for the indicated time points, followed by incubation with cell counting kit-8 (CCK-8) substrate (Dojindo) for 1 h at 37 °C. The absorbance was examined at 450 nm by the GloMax Explorer (Promega).

### Flow cytometry

Cells were treated with cisplatin (8 µM) and/or Biochanin A (100 µM) for 48 h. Cells, together with the supernatants, were collected and subjected to apoptosis analysis using the FITC Annexin-V/PI apoptosis detection kit (BD Biosciences), according to the manufacturer’s protocols. The percentage of apoptotic cells was assessed by flow cytometry. Each experiment was performed in triplicates.

### Immunofluorescence staining

Cells were treated with cisplatin (8 µM) and/or Biochanin A (100 µM) for the indicated time points and fixed in 4% paraformaldehyde, followed by incubation with γH2AX antibody (9718 S, CST) at 4 °C overnight. Cells were then incubated with DyLight 594-conjugated secondary antibody for 1 h and stained with DAPI (1 µg/ml) for 3 min. The labeled nuclei were detected by Confocal FV1000 (Olympus). The percentage of cells with ≥ 10 foci was quantified. At least 100 cells were counted per well.

### Tumor xenograft

The BALB/c nude mice (6 ~ 8-week-old) were injected with A549 cells (1 × 10^6^ cells/100 µl PBS) and separated randomly into four groups, followed by treatment with DMSO, Biochanin A (oral gavage, 100 mg/kg), cisplatin (intraperitoneal injection, 5 mg/kg) and Biochanin A + cisplatin, respectively.

### Immunohistochemistry

Tumor tissues were fixed in formalin and then embedded in paraffin. Tumor sections were stained with the appropriate antibodies (Table S2) using the Envision Kit (Dako). Images were taken with an Olympus IX53 (Olympus). Six fields were randomly selected, and the number of positive cells and the total number of tumor cells in each field were counted. The number of positive cells was then divided by the total number of tumor cells to obtain the percentage of positive cells in each field. The IHC score was calculated by combining the quantity score (the percentage of positively stained areas) with the staining intensity score. The quantity score ranges from 0 to 4: 0, no immunostaining; 1, 1–14% of the areas are positive; 2, 15–49% of the areas are positive; 3, 50–74% of the areas are positive; and 4, ≥ 75% of the areas are positive. The staining intensity was scored as follows: 0 (no color), 1 (light yellow), 2 (light brown), 3 (brown), and 4 (dark brown). The score for each tissue was calculated by summing the intensity and quantity scores, and the range of this calculation was therefore 0–8.

### Immunoprecipitation assay

Cell lysates were incubated with anti-ZEB1 antibody and protein G Dynabeads (Invitrogen) overnight at 4 °C, followed by three washes with cold PBS buffer. The immunoprecipitates was then subjected to immunoblotting analysis.

### Ubiquitination assay

Cells were transfected with HA-ubiquitin plasmids, followed by treatment with MG132 and Biochanin A. Cell lysates prepared in RIPA buffer with protease and phosphatase inhibitors were subjected to immunoprecipitation with an anti-ZEB1 antibody to detected the ubiquitination level of ZEB1 protein.

### Statistical analysis

SPSS 23.0 software and GraphPad Prism 7.0 were used for the statistical analysis. The data are presented as the means ± SD and represent at least three independent experiments. Student’s *t*-test was used for the unpaired observations. One-way analysis of variance (ANOVA) was used to compare means between treatment groups. A *P*-value < 0.05 was considered to have significant difference.

## Supplementary Information

**Additional file 1.**

## Data Availability

The datasets generated during and/or analysed during the current study are available from the corresponding author on reasonable request.
