# Supplementary material for: Biochanin A inhibits lung adenocarcinoma progression by targeting ZEB1
Source: Discov Oncol. 2022 Dec 13;13:138. doi: 10.1007/s12672-022-00601-2 (PMC9748019; doi:10.1007/s12672-022-00601-2)
Supplement: Supplementary file 1 — Additional file 1. [file 12672_2022_601_MOESM1_ESM.docx]

**Supplementary Information for**

**Biochanin A inhibits lung adenocarcinoma progression by targeting ZEB1**

Jianjun Li^1,2*^, Yaqi Kou^1*^, Xiaohan Zhang^1*^, Xuechun Xiao^1^, Yang Ou^1^, Lixia Cao^1^, Min Guo^1^, Chunchun Qi^1^, Zhaoyang Wang^1^, Yuxin Liu^1^, Qiuying Shuai^1^, Hang Wang^1#^, Shuang Yang^1,3#^

^1^Tianjin Key Laboratory of Tumor Microenvironment and Neurovascular Regulation, Medical College of Nankai University, Tianjin 300071, China

^2^ Department of Pulmonary and Critical Care Medicine, The First Affiliated Hospital of Soochow University, 215006 Suzhou, China

^3^Institute of Transplantation Medicine, Nankai University, Tianjin 300071, China

^*^These authors equally contribute to this study.

^#^Corresponding authors: Shuang Yang; Medical College of Nankai University; 94 Weijin Road, Tianjin 300071, China; Email: [yangshuang@nankai.edu.cn](mailto:yangshuang@nankai.edu.cn); Hang Wang; Medical College of Nankai University; 94 Weijin Road, Tianjin 300071, China; Email: [wanghang@nankai.edu.cn](mailto:wanghang@nankai.edu.cn)

**This file includes:** Figure S1 to S5, Tables S1 to S2.


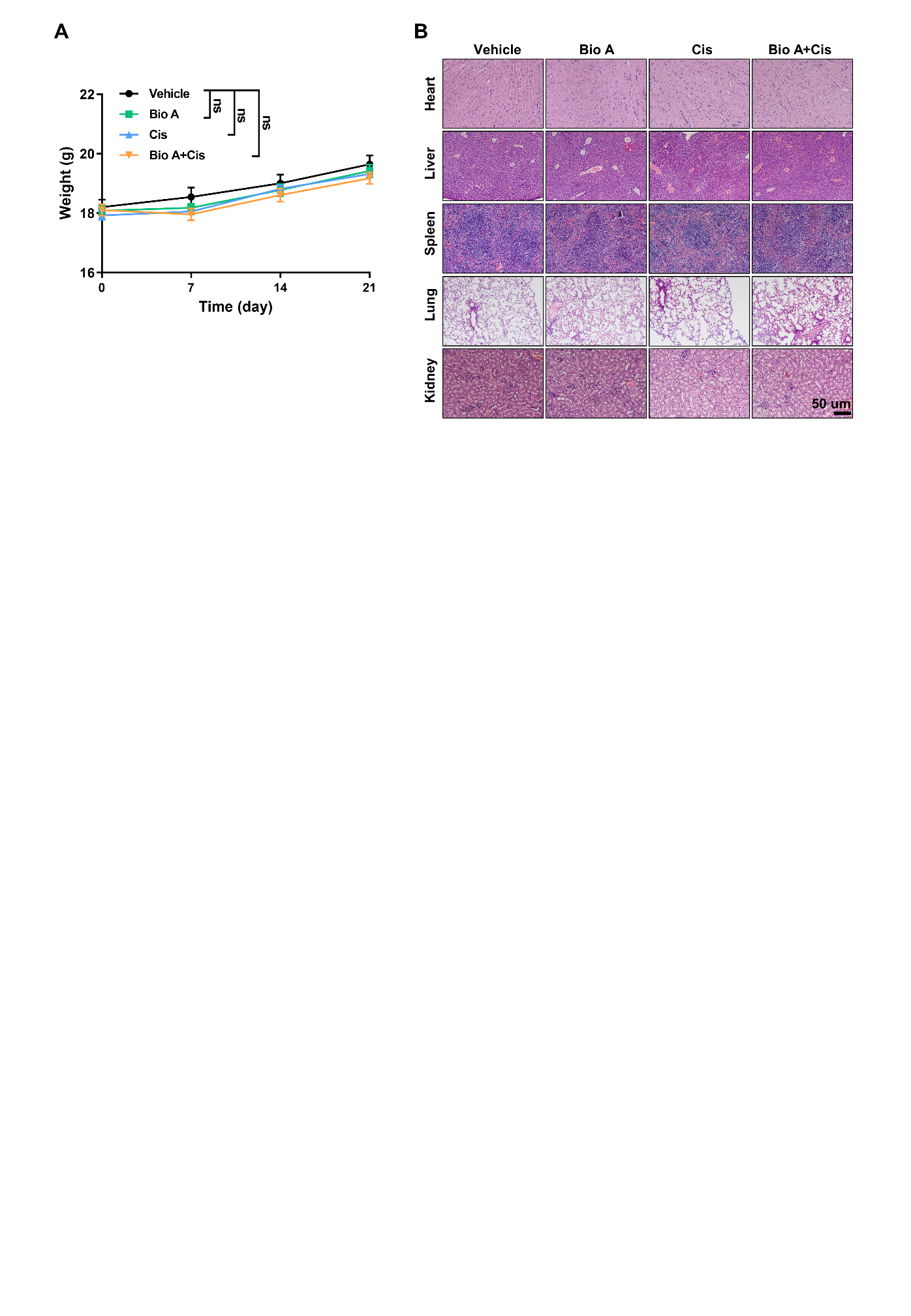


**Figure S1.** Biochanin A has no significantly adverse effects. (**A**) Animal weight of mice by treatment with Biochanin A and/or cisplatin were measured. (**B**) H&E staining was performed on heart, liver, spleen, lung, and kidney tissues from mice by treatment with Biochanin A and/or cisplatin.


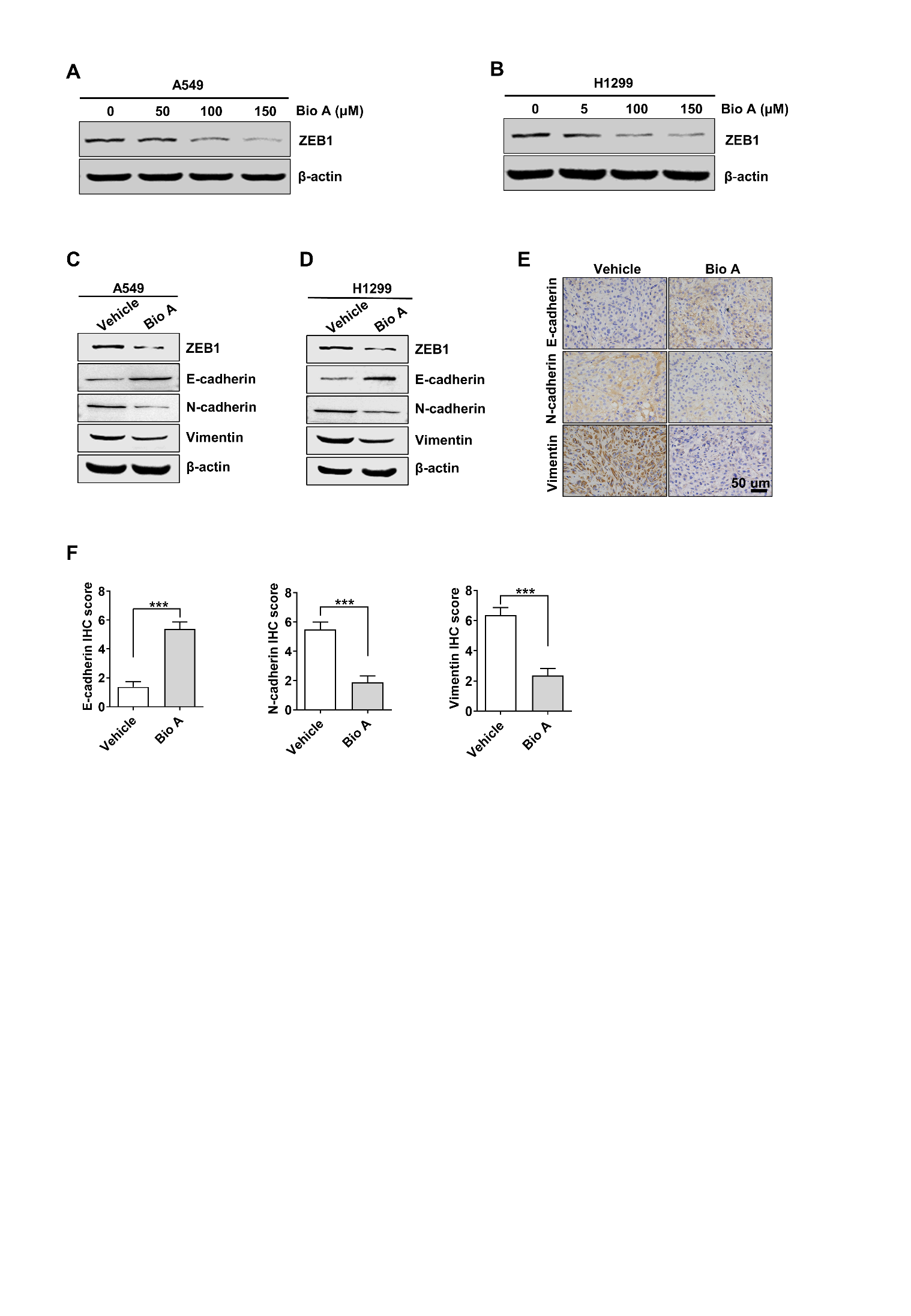


**Figure S2.** Biochanin A downregulates the expression of ZEB1 protein. (**A-B**) Immunoblotting analysis of ZEB1 expression in A549 (**A**) and H1299 (**B**) cells by treatment with different concentrations of Biochanin A. (**C-D**) Immunoblotting analysis of EMT markers in A549 (**C**) and H1299 (**D**) cells by treatment with Biochanin A. (**E-F**) Immunohistochemistry staining (**E**) and quantification (**F**) of EMT markers in A549-expressing tumors by treatment with Biochanin A. ****P* < 0.001 vs respective control by an unpaired Student’s *t*-test.


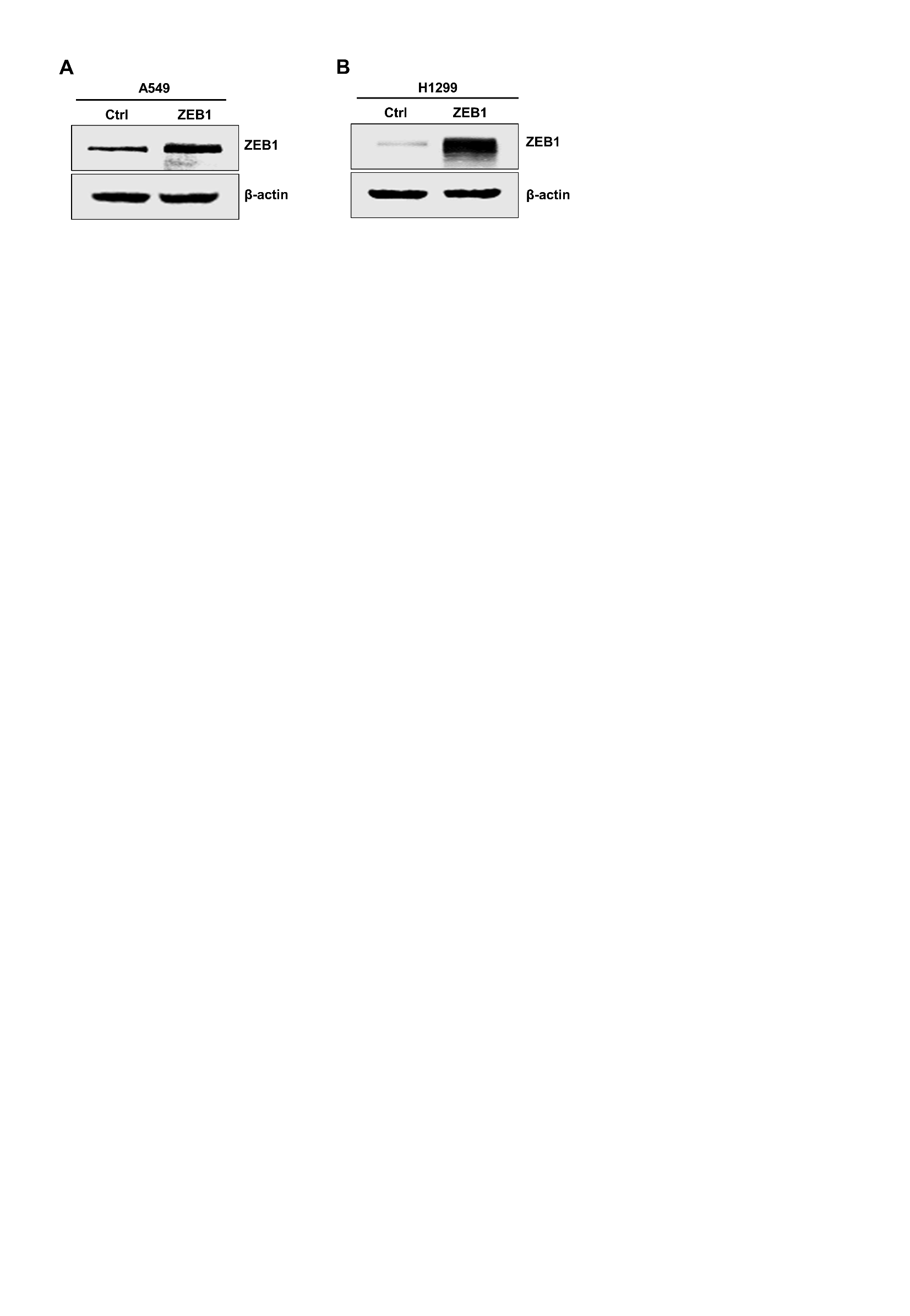


**Figure S3.** Construction of ZEB1-expressing stable cell lines. (**A-B**) Validation of ZEB1 expression in ZEB1/A549 (**A**) and ZEB1/H1299 (**B**) cells.


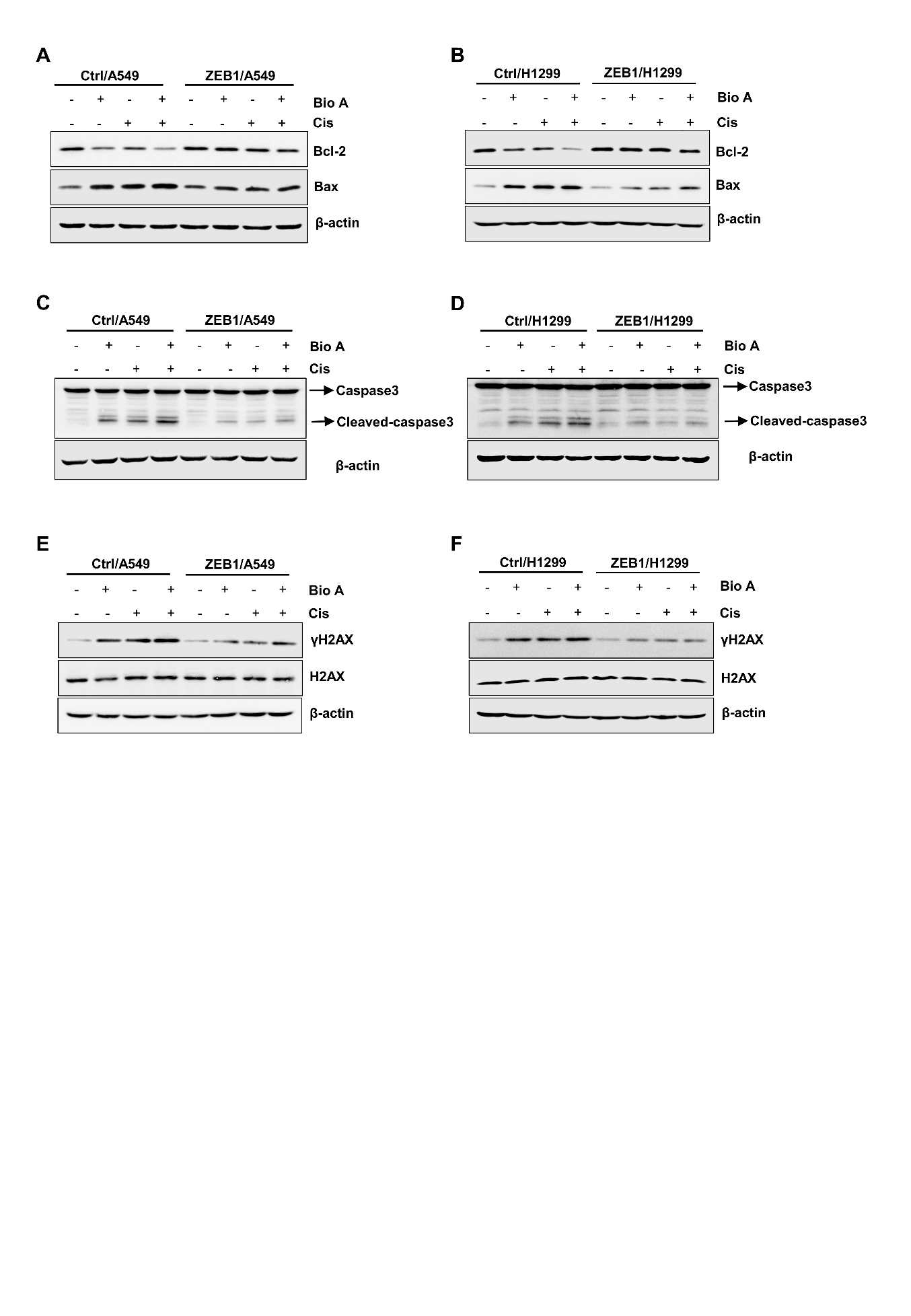


**Figure S4.** Biochanin A chemosensitizes lung adenocarcinoma through downregulation of ZEB1 *in vitro*. (**A-B**) Immunoblotting analysis of Bcl-2 and Bax in ZEB1-expressing A549 (**A**) and H1299 (**B**) cells by treatment with Biochanin A and/or cisplatin. (**C-D**) Immunoblotting analysis of cleaved-caspase3 in ZEB1-expressing A549 (**C**) and H1299 (**D**) cells by treatment with Biochanin A and/or cisplatin. (**E-F**) Immunoblotting analysis of γ-H2AX and H2AX in ZEB1-expressing A549 (**E**) and H1299 (**F**) cells by treatment with Biochanin A and/or cisplatin.


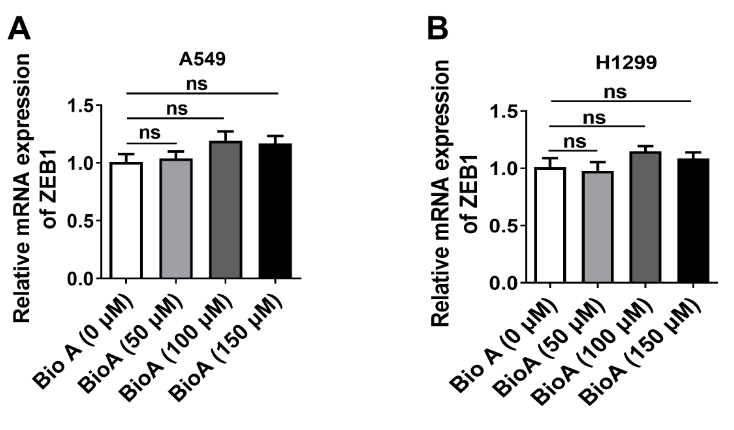


**Figure S5.** Biochanin A does not alter the mRNA expression of ZEB1. (**A-B**) Relative mRNA expression of ZEB1 in A549 (**A**) and H1299 (**B**) cells by treatment with different concentrations of Biochanin A.

**Table S1. Primers used in this study.**

| **Construction of Zeb1 expression vector** | |
| --- | --- |
| ZEB1 forward | 5’-ATGGCGGATGGCCCCAGG‑3’ |
| ZEB1 reverse | 5’- TTAGGCTTCATTTGTCTTTTC‑3’ |
| **Quantitative RT-PCR** | |
| human ZEB1 forward | 5’-CAGCTTGATACCTGTGAATGGG-3’ |
| human ZEB1 reverse | 5’-TATCTGTGGTCGTGTGGGACT-3’ |
| human GAPDH forward | 5’-GGAGCGAGATCCCTCCAAAAT-3’ |
| human GAPDH reverse | 5’-GGCTGTTGTCATACTTCTCATGG-3’ |

**Table S2. Antibodies used in this study.**

| Antibodies | Species | Application | Manufacturer | Catalog No. | Dilution |
| --- | --- | --- | --- | --- | --- |
| anti-ZEB1 | Rabbit | IHC | Abcam | ab87280 | 1:400 |
|  | Rabbit | IB | Proteintech | 21544-1-AP | 1:1000 |
| anti-β-actin | Mouse | IB | Santa Cruz | sc-47778 | 1:1000 |
| anti-HA-Tag | Rabbit | IB | Cell Signaling Technology | 3724S | 1:1000 |
| anti-Bax | Rabbit | IB | Cell Signaling Technology | 14796S | 1:1000 |
| anti-Bcl-2 | Mouse | IB | Cell Signaling Technology | 15071S | 1:1000 |
| anti-TWIST | Rabbit | IHC | Cell Signaling Technology | 69366S | 1:1000 |
| anti-SNAIL | Rabbit | IHC | Cell Signaling Technology | 3879S | 1:1000 |
| anti-Vimentin | Rabbit | IB | Cell Signaling Technology | 5741S | 1:1000 |
|  | Rabbit | IHC | Cell Signaling Technology | 5741S | 1:400 |
| anti-N-cadherin | Rabbit | IB | Cell Signaling Technology | 13116S | 1:1000 |
|  | Rabbit | IHC | Cell Signaling Technology | 13116S | 1:100 |
| anti-E-cadherin | Mouse | IB | Cell Signaling Technology | 14472S | 1:1000 |
|  | Mouse | IHC | Cell Signaling Technology | 14472S | 1:100 |
| anti-Cleaved- caspase3 | Rabbit | IB | Cell Signaling Technology | 14220S | 1:1000 |
|  | Rabbit | IHC | Cell Signaling Technology | 9664T | 1:2000 |
| anti-Ki67 | Mouse | IHC | Cell Signaling Technology | 9449S | 1:400 |
| anti-H2AX | Rabbit | IB | Cell Signaling Technology | 7631S | 1:1000 |
| anti-γH2AX | Rabbit | IB | Cell Signaling Technology | 9718S | 1:1000 |
|  | Rabbit | IF | Cell Signaling Technology | 9718S | 1:400 |
|  | Rabbit | IHC | Cell Signaling Technology | 9718S | 1:400 |

IHC: Immunohistochemistry; IB: Immunoblotting; IF: Immunofluorescence
